# Characterization of Deposits Formed in Gas Engines Fuelled by Coal Mine Methane

**DOI:** 10.3390/ma16062517

**Published:** 2023-03-22

**Authors:** Izabela Konkol, Jan Cebula, Lesław Świerczek, Jan Sopa, Janusz Sopa, Adam Cenian

**Affiliations:** 1 Physical Aspects of Ecoenergy Department, Institute of Fluid-Flow Machinery, Polish Academy of Sciences, Fiszera 14 Street, 80-231 Gdańsk, Poland; 2 Partner Hs Halina Sopa, Polish Company, Barcioka 27a Street, 44-282 Czernica, Poland

**Keywords:** deposits, microanalysis, chemical analysis, failure, mine gas engine

## Abstract

The main purpose of this research was to determine the chemical composition of deposits in gas engines fuelled by coal mine methane (CMM), as well as its dependence on the place of collection. This composition was compared with that for deposits formed in biogas-powered engines. It was also found that the chemical composition of deposits varies depending on the place of their formation in the engine and on the gaseous fuel used. The dominant mineral deposits found in gas engines fuelled by CMM contained Ca, Zn, P, and S, which originate from oil additives. The Al, Cr, Cu, Ti, and Fe elements present in the tested samples are related to the wear of the engine under normal operation. The remaining trace elements can originate as impurities from the air.

## 1. Introduction

Fossil fuels are the main fuels used in different economic sectors, such as transport, industry, agriculture, and energetics [1]. Natural gas, often extracted from oil fields, is a mixture of light hydrocarbons naturally occurring in rocky formations beneath the earth’s surface. As its main component is methane, natural gas has a high calorific value per unit of volume and low carbon atom content [2], which fulfil the growing global demand for clean and relatively cheap energy [3].

The use of natural gas also has a greenhouse gas (GHG) emission-benefit because it emits approximately 30% less carbon dioxide from its combustion due to the high H/C ratio compared to gasoline or diesel fuel on the same energy content basis [4,5,6].

Coalbed methane (CBM) is a primary type of unconventional natural gas that is inherent to coal deposits; it is formed during biomass coalification. CBM refers to the methane emitted in the underground-mining process, either from coal seams being mined or from other methane-containing formations near the mined coal seam [7,8,9,10,11]. Coal mine methane (CMM) is a general term for methane mixtures, which must, for safety reasons, be extracted mainly during and after mining operations. These gases show high variability in flow rate and composition [7,9,12]. Coal mine methane typically contains from 25% to 60% of methane [13]. The CMM gas capture by wells initially contains even up to 95% (by volume) methane but has production rate and quality declines through time [9,14]. Methane-related accidents cause the majority of disasters in underground-mining and pose a great danger (e.g., methane explosions) [10]. In addition to improving mining safety, CMM use leads to environmental benefit, i.e., it provides a source of relatively clean energy and raw materials [10]. It also reduces GHG emissions because the greenhouse effect caused by uncombusted methane is 21 times higher than the CO_2_ released by its combustion [8,10]. Due to the high methane content, extracted CMM can be used as a chemical feedstock or fuel for energy by direct combustion in internal combustion engines to generate electricity and heat, or it is even more efficiently used for polygeneration, i.e., to produce electricity, heat, and cold [9,11,13]. In addition to methane, CMM gas contains CO_2_ and some trace impurities. Methane itself is chemically stable and does not normally interact with engine parts; however, trace impurities (e.g., chlorides or dusty particles), often present in CMM, can cause deleterious effects on those parts [9,15], including increased corrosion. 

As mentioned above, the composition of the gaseous fuel may adversely affect the functional properties of spark-ignition gas engines. Components sensitive to contaminants present in gaseous fuel include, among others, valves in engine heads. The role of the inlet valve is the introduction of the appropriate fuel and oxygen mixture into the combustion chamber. Exhaust valves, on the other hand, are designed to direct exhaust gasses from the combustion chamber to the surrounding atmosphere [3,16,17]. The proper operation of the valves translates directly into engine performance, such as power, torque, fuel consumption, and exhaust emissions. The inevitable problems of internal combustion engines are wear, tear, and valve failure, which, in turn, leads to reduced efficiency, downtime, and high maintenance costs [17,18]. The main causes of failure include overheating and loss of material strength at high temperature, oxidation, fretting, galling, and impact load [19]. It is worth mentioning that exhaust valve failure can result from both lubricant (too much ash) and non-lubricant factors, as well as from valve recession itself [3]. The deposition of solid residues on the surface of the valves can cause loss of their tightness, strength, and excessive wear, which all manifest directly as reduced efficiency of the engine.

Another common problem with both CMM and natural-gas-powered engines is spark plug erosion and consequent failure. It is estimated that the spark-plug lifetime of gas engines used in energetics could reach up to 8000 h, but their wear is often observed already after 1000–4000 h. This results in a loss of efficiency and the need for frequent, costly downtime, related to their replacement [19,20]. 

In addition to excessive wear of spark plugs in spark-ignition engines, the deposition of mineral solids is observed on various parts of the engine, e.g., cylinder heads, pistons, exhaust manifold, etc. Sources of deposit formation include incomplete fuel combustion, its properties, and the leakage of lubricating oil into the combustion chamber caused by thermal cracking of engine components [3]. The deposits affect the quality of the injection process, engine power, fuel consumption, heat transfer efficiency and, consequently, GHG emission [21,22]. However, the process of deposit formation is not fully known and requires further intensive research. 

M.I. Khan et al. stated that the most important parameter influencing the formation of deposits is the engine surface temperature. Compounds contained in contaminated fuel as well as in lubricating oil can form low-melting-point salts under high temperature conditions. It is estimated that the exhaust gas temperature in the vicinity of the exhaust valve reaches 600 to 950 °C [3,23]. Operating temperatures lower than 700 °C can cause salt deposition on valves or other parts of the combustion chamber. Actions such as increasing engine speed, load, and coolant temperature reduce deposit formation due to increased operating temperatures. After reaching the temperature of the salt’s melting point, they are partly carried away by the exhaust gases [3,23]. However, due to the insulating properties of deposits and changes in engine surface temperatures, the balance between the salt’s formation and removal is established for each operating temperature [21]. Moreover, higher operating temperatures may contribute to the degradation of lubricant and release mineral additives. This phenomenon has been described in the work of Mathai et al. [24]. The authors observed a higher deposit formation rate on a piston engine fuelled with compressed natural gas blended with 18% hydrogen when compared to gas without hydrogen addition. This resulted from higher operating temperatures. 

Lin et al. studied J-type spark plugs with Ni-based alloys, which were exposed to combustion in a Caterpillar 770 ekW natural gas engine [20]. The tip insert for the centre electrode was Ir, whereas the ground electrode was a Pt-W based alloy. Worn spark plugs were characterized after 2020 and 4386 h and then compared with new ones. Researchers noted a significant amount of Ca on the spark plugs’ bodies, but there was no significant difference in Ca intensity between 2020 and 4386 working hours of the tested spark plugs. On the nickel-base alloy, glassy phases with Ca, Zn, P, and S, were found. Furthermore, a significant quantity of Ca in the oxide phase was found. Calcium, known as an amorphous phases modifier, can cause a significant decrease in the viscosity and softening temperatures. In combination with the oxides present on the spark plug’s surface, it can contribute to the erosion of base electrode and spark plug tip inserts during sparking at elevated temperatures [20]. 

Lubricating oil mainly consists of base oil and additives. Additives improve its performance or properties such as maintaining engine cleanliness, antioxidation, and reduced friction and wear [15]. Elements such as Ca, O, P, S, Mg, B, and Zn are common in engine oil (zinc dithiophosphate, calcium sulfonates, etc.) and are, presumably, partial residues from evaporated oil [17,25]. Zinc and Phosphorus exist in zinc dialkyl dithiophosphate that is used to protect the components from abrasion and oil oxidation. Calcium-containing oil additives are used as a detergent agent to control rust and the accumulation of plastic material in the engine. Elements such as Fe, Cu, Al, Mg, Mn, Cr, and Pd are formed as a result of the abrasion of engine materials [21]. Therefore, the presence of the above-mentioned elements in the deposits is most often caused by the thermal degradation of the lubricating oil, as well as the partial wear of individual engine components.

During the combustion of contaminated gaseous fuel and oil entering the combustion chamber, it was shown that the deposits formed on the valve seat, face, and stem consisted of sulphates, phosphates, and metal oxides [3,26,27]. Khan et al. [3] analysed deposits formed on the valve surface using XRD technique. They identified anhydrite (CaSO_4_), hydroxyapatite (Ca_5_(PO_4_)_3_OH), zincate (ZnO), and calcite (CaCO_3_). Hydroxyapatite is a form of enamel that is difficult to remove. As a result of the mentioned compounds’ depositions, intergranular corrosion was observed, leading to the valve sealing face and its other parts burning out. 

A similar phenomenon was observed in the case of gas engines powered by landfill gas; however, volatile methyl siloxane had the largest share in the formation of deposits on engine parts [28,29]. Metals such as Se, Te, Hg, Pb, As, Sn, Sb, Bi, Cl, S, Mg, Cu, Si, Ca, P, and Zn have been found in analysed deposits [28,29,30,31]. Consequently, anhydrite (CaSO_4_) and akaganeite (Fe^3+^O(OH)) were found on the exhaust valve, the cylinder, and the piston heads. Gypsum (CaSO_4_·2H_2_O) was detected in the cylinder head deposit. Iron Sulphate (FeSO_4_) was detected in the exhaust valve deposit. Ca, S, and Fe elements, which represent a substantial proportion of the deposit, have been involved in the formation of crystal compounds as detected in the XRD pattern of all deposit samples [28]. 

Deposits collected from various parts of the engine powered by biogas from a waste-water treatment plant were characterized by a similar composition. XRD analysis confirmed oxide forms such as SiO_2_, Bi_2_O_3_, CaO, P_2_O_5_, and ZnO [32]. However, the highest contents found were silicon, calcium, phosphorus, sulphur, and zinc [32], which were also confirmed by Stanuch et al. [25]. 

The analysis of the presented literature confirmed that the composition of deposits in gas engines was mainly affected by the type of fuel used, engine wear, and leakage of lubricating oil into the combustion chamber. However, by analysing the available literature, there was a lack of information about deposit characterization formed in CMM gas engines; only one source characterized the deposits formed in a natural gas engine and, even then, only from the spark plugs. The other papers confirmed that deposits were formed, but their chemical composition was not analysed. It was shown that they affected the lubricating properties, creating an insulating layer, which contributed to engines’ mechanical damage. It was believed that deposits were formed as a result of the reaction of impurities and lubricating oil. In addition, there were more papers on the analysis of deposits in engines powered by landfill gas or biogas than there were for CMM.

This paper presents the results of deposits’ characterization formed in gas engines fuelled by CMM. Samples of deposits were collected from different parts of a gas engine during field service for a better understanding of deposit formation processes during engine operation. The analysis of composition enabled the prediction of the wear of individual engine components, as well as the estimation of the quality of the gaseous fuel used. Such analysis could be helpful in appropriate selection of a gas purification system in order to reduce downtime and engine servicing.

## 2. Materials and Methods

**Figure 1:**
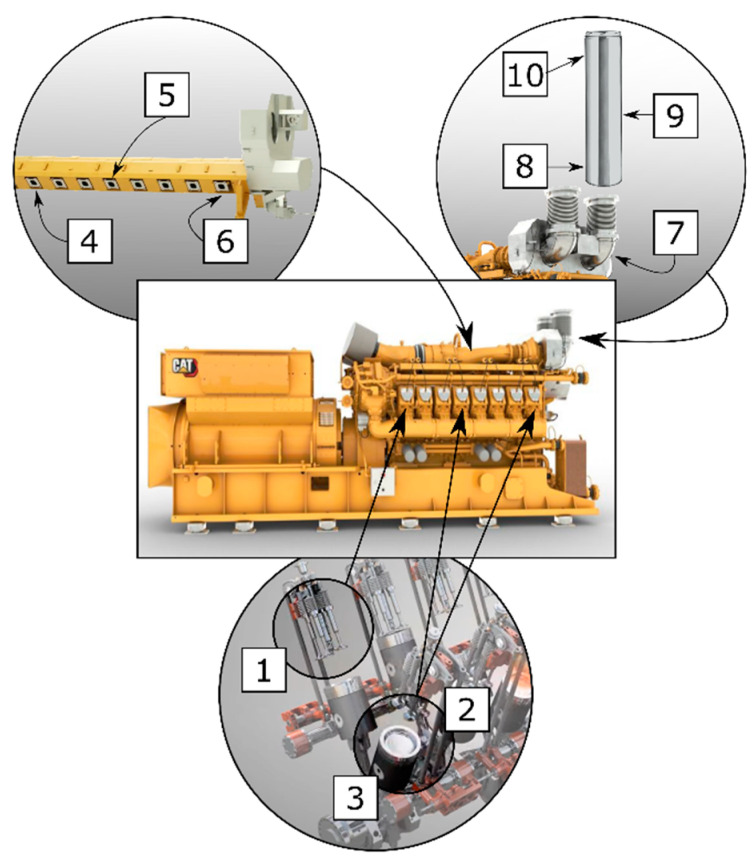
Engine scheme with deposits sampling points: engine combustion chamber: 1—cylinder head; 2, 3—pistons; exhaust manifold: 4—first cylinder; 5—fourth cylinder; 6—eight cylinder, collector: 7—exhaust pipe after compressor and cooler; 8–10—roof chimneys.

Deposit samples were collected from various parts of the gas engine (a Caterpillar 850 kW) such as combustion chamber, exhaust manifold, and flue during technical service. The sampling locations for all samples are shown in Figure 1. 

**Figure 2:**
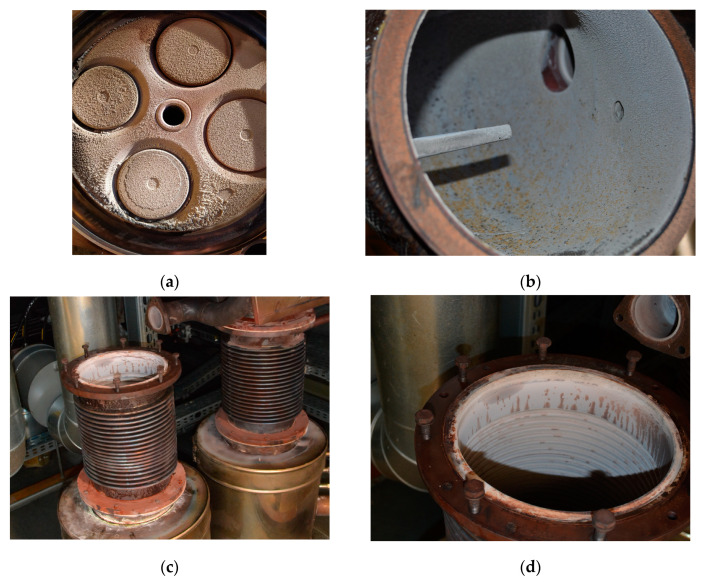
Deposits formed on different parts of gas engine: (**a**) cylinder head with valves; (**b**) exhaust pipe in front of the chimney inlet; (**c**,**d**) roof exhaust pipe/flue.

Figure 2 shows photos of deposits formed on selected engine parts during sampling. The composition of the CMM gas used in the current exploitation was variable and included: CH_4_—25–60%; CO_2_—1–6%; CO—0.1–0.4%; O_2_—7–17%; and N_2_—4–40%. Additionally, on the engine air intake filter, contaminants such as hydrogen sulphide, formaldehyde, phosphine, nitrous oxide, (E)-1-Ethanesulphinyl-2-phenylethane, acridine-9-thione, thiophene, and phenanthrene were found. The hydrogen sulphide concentration was measured using a portable gas analyser (GA5000, Geotech, Manchester, UK), while organic compounds were determined by gas chromatography coupled with a mass spectrometer (GC-MS) (Thermo Scientific, GC 2010, Waltham, MA, USA).

Samples were scraped with a sharp chrome-nickel steel knife from places where the layer of deposits was the thickest, whereas some of the deposits were loosely connected to the surface and could be easily sampled. 

The morphology of the samples was studied with scanning electron microscopy (SEM), operated with a secondary electron detector in high vacuum mode at an accelerating voltage of 10–20 kV (FEI Quanta FEG 250, Thermo Fisher Scientific, Waltham, MA, USA). The deposits were coated with gold to increase the conductivity of the samples before they were analysed by the SEM. The samples were mounted on double-sided carbon tape. Quantitative and qualitative area analysis was performed using energy dispersive spectroscopy (EDS) on EDAX Genesis APEX 2i (Ametek, Berwyn, PA, USA) with an Apollo X SDD spectrometer at an accelerating voltage 30 kV, operating at 132 eV resolution at 5.9 KeV. The size of the analysed particles was in the range of 0.5–2.5 µm (average was 1.12 µm).

Engine lubricating oils (Pegasus 1005/40) were analysed using inductively coupled plasma–mass spectrometry (ICP-MS) (Agilent 7700s, ICP-MS, Santa Clara, CA, USA).

## 3. Results and Discussion

**Figure 3:**
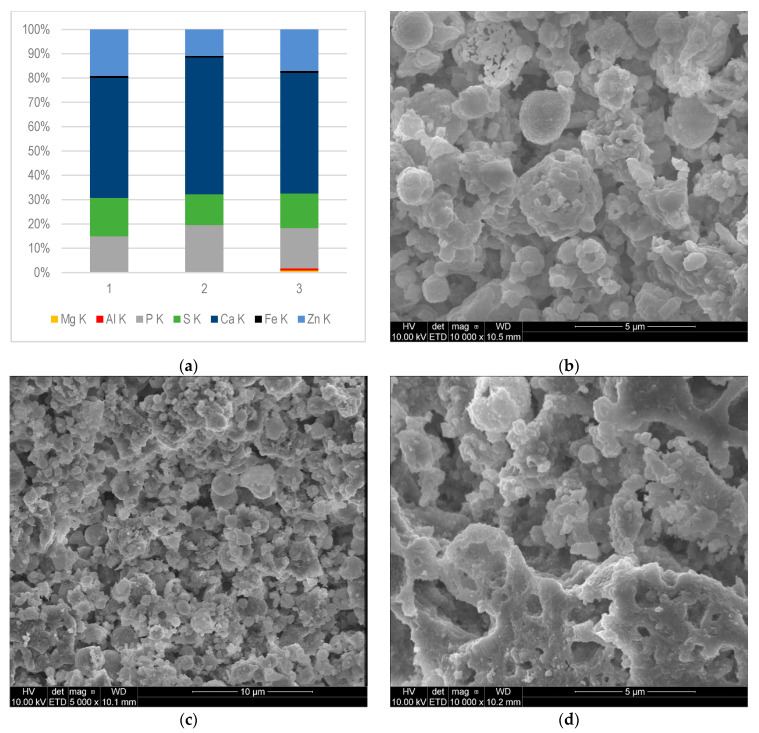

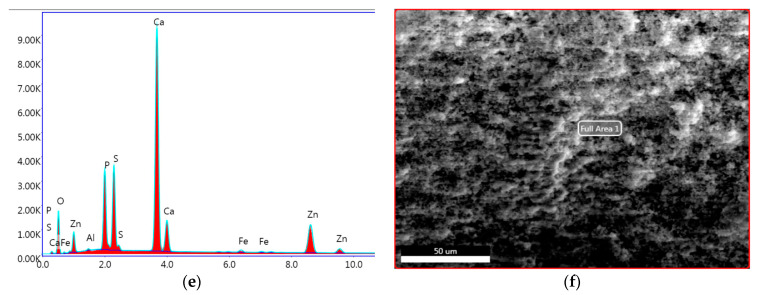
SEM-EDS composition microanalysis of deposits from the combustion chamber: (**a**) samples 1, 2, and 3 determined by mass fraction (%), K—the excited energy level of an atom; (**b**–**d**) morphological characteristics of sample 1, 2, and 3, respectively; (**e**) EDS spectrum (x axis—Energy (keV), y axis—Intensity (cps)) of (**f**) selected area for sample 1.

**Figure 4:**
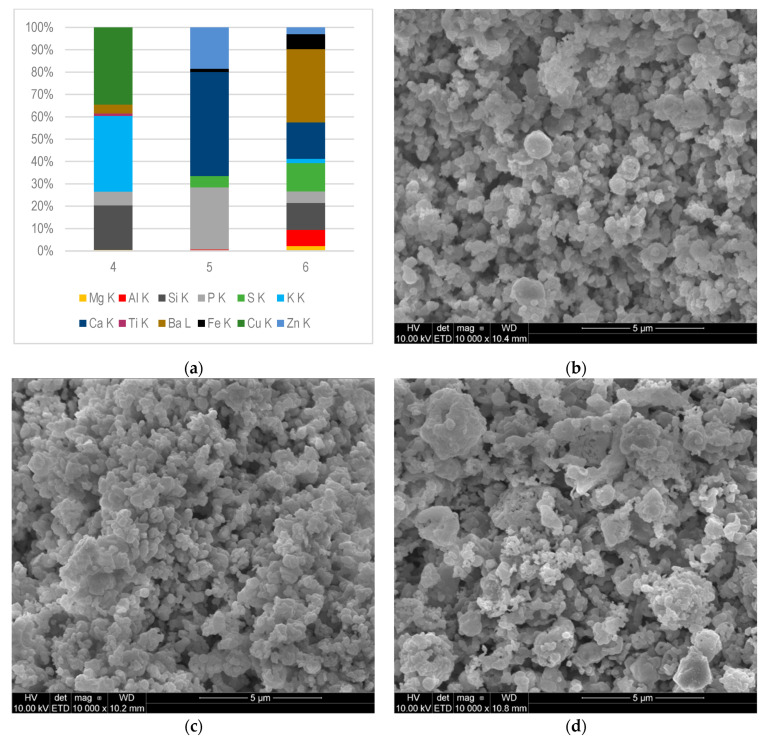
Deposits collected from exhaust manifold (samples 4, 5 and 6): (**a**) chemical composition determined by SEM-EDS microanalysis–mass fraction (%); K and L—the excited energy level of an atom; (**b**–**d**) morphological characteristics of deposit samples 4, 5, and 6.

**Figure 5:**
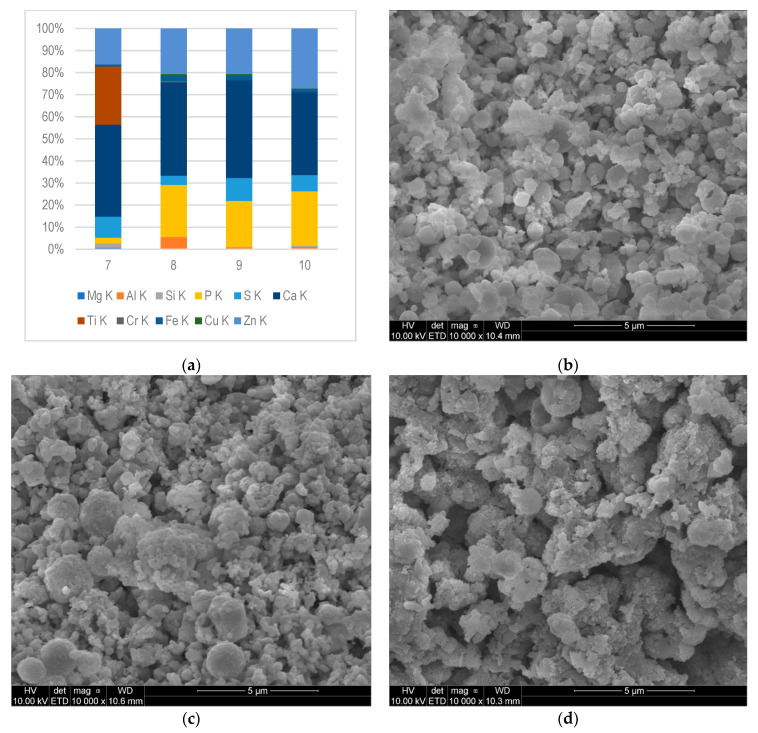
Deposit samples collected from exhaust pipe and chimney: (**a**) the chemical composition determined by SEM-EDS microanalysis–mass fraction (%), K and L—the excited energy level of an atom; (**b**–**d**) Morphology of the deposits collected from exhaust pipe in front of chimney (7) and chimneys (8, 9, 10).

The solid deposits from different parts of the gas engine fuelled by CMM were subjected to elemental composition analysis. Elemental composition in mass fractions (%) of deposits, and SEM photos are presented in Figure 3, Figure 4 and Figure 5. 

Figure 3a,e,f shows the SEM-EDS analysis results of samples collected from the combustion chamber—the cylinder head (1) and individual pistons (2, 3). 

The composition of deposits sampled from the combustion chamber was very similar. The largest mass contribution was observed for Ca (49–56%). In analysed deposits, the contents of P, Zn, and S were in the ranges 15–20%, 11–19%, and 13–16%, respectively. Fe content was found in all tested samples (0.64–0.81%), while Al was present (0.26–0.76%) in samples 1 and 3. The presence of Mg (0.93%) was found only in deposit (sample) 3.

Based on the analysis of the presented results, it can be seen that the elemental composition of the deposits was similar, due to similar temperature and pressure conditions inside the combustion chamber. The presence of Fe and Al in all samples may indicate the partial wear of the engine’s components. These elements, in the form of metal particles or organometallic compounds, during the engine’s operation pass into the lubricating oil and then into deposits [33]. Zn, Ca, and P came mainly from additives that improved engine oil performance; therefore, they formed the largest share of deposits [34].

Figure 4a shows the results of SEM-EDS analysis of the deposits collected from different parts of the exhaust manifold: 4—first cylinder exhaust; 5—fourth cylinder exhaust; and 6—eighth cylinder exhaust. Figure 4b–d shows the morphology of the samples. As it can be seen, although the samples were taken from the same engine element, their compositions were significantly different. Phosphorus was found in all tested samples collected from the exhaust manifold. The weight fraction of P in samples 4 and 6 was very similar and amounted to 6.1% and 5.2%, respectively. In sample 5, the share of phosphorus differed significantly and amounted to 28%. Similarly, the largest share in the analysed sample 5 had Ca (47%), whereas Zn content was 18%. As mentioned, P, Ca, S, and Zn are common components of lubricating oil [34]. Moreover, the composition variabilities of the elements mentioned in samples 4, 5, and 6 may indicate a different degree of wear of the individual components of the combustion chamber. It can be noted that deposit sample 5 contained the largest amounts of P, Ca, and Zn, which were derived from engine oil leaks and their thermal degradation. In sample 4, Ca, Zn, and S were not observed, while the contents of these elements in sample 6 were 16%, 18%, and 13%, respectively. The reduced content of Ca and Zn in the above-mentioned samples may indicate a greater tightness of the combustion chamber elements, as well as salt transport to further parts of the engine exhaust system.

Both, samples 4 and 6 contained elements such as Mg, K, and Ba, in the ranges 0.46–2.2%, 1.9–34%, and 3.9–33%, respectively. As shown in the work by Zali et al., the source of elements such as Mg and Ba may be additives to lubricating oils. The presence of Si, Fe, Al, Ti, Ba, and Cu in deposits may have their sources both as impurities delivered with fuel and air as well as engine components wear [34].

Sample 6 was the most qualitatively different, containing elements such as Zn, Fe, Ba, Ca, K, S, P, Si, Al, and Mg. It can, therefore, be assumed that the quantity and quality of the deposits varied along the exhaust manifold. Konkol et al. [29] also observed that the composition of the deposits taken from different parts of the engine may differ. 

Figure 5a shows the results of SEM-EDS analysis of the deposits collected from different places of the exhaust pipe and flue: sample 7—from exhaust pipe in front of the flue, while sample 8 was collected from the beginning; 9—from the middle part; and 10—from the terminus (outlet) of the exhaust, respectively. Figure 5b–d present the morphologies of samples 8, 9, and 10.

Due to the similar distribution of temperatures in the flue, the composition of deposits collected from its three different places (samples 8–10) was not significantly different. Al, P, S, Ca, Fe, and Zn were found in all analysed deposits. It was shown that Ca made up the largest share of the mass of deposits (from 38% to 44%). The second dominant element was Zn, its contents ranged from 16% to 27%. In the case of the average content in all deposits, the third dominant element was P (18%). As mentioned, Zn, Ca, S, and P are components of lubricating oil. In addition, the presence of Al (0.34% to 5.5%) and Fe (1.1% to 2.3%) was found. The aforementioned elements in the deposits had their origins from worn moving parts of the gas engine.

Similar contents of the Cu and Cr elements, which form volatile compounds [35] at high temperatures, were recorded in the samples from the flue. These elements, indicating partial wear and corrosion of bearings or valve surfaces, settled inside the flue, despite the decrease in exhaust gases temperature.

It is worth noting that the deposits in sample 7 differed in quantitative and qualitative composition from the others. In addition to the main deposit components such as P, Ca, S, and Zn, elements such as Si (1.7%), Mg (0.68%), Cr (0.56%), Ti (26%), and Fe (0.86%) were also found. The high content of Ti, in parallel with the occurrence of Cr and Fe, may indicate that they were wear elements. However, a definitive explanation of the highest Ti content among all analysed samples was difficult and required further research. The occurrence of Ti may be related to the condensation of Ti-containing compounds as a result of exhaust gas cooling in the flue inlet pipe, a change in the gas flow velocity, or a change in the shape of the internal element. Titanium can be used also as an additive to engine oils [25]. It can also come from alloys [3].

Mineral nanoparticles, which were formed under the influence of high pressure and temperature, hit the hot surface of the engine and then accumulated there, forming a flat thin layer. Then, other particles accumulated irregularly, forming layers on the initially formed surface. Figure 3b–d, Figure 4b–d, and Figure 5b–d show the top surfaces of the deposits, which depict the shapes of the particles clearly. They assumed a cauliflower shape. The embossed particles of various sizes and shapes were superimposed on each other irregularly, as seen from the images. A similar cauliflower shape was observed by Östürk and Sevimoğlu [28].

The elemental analysis of lubricating oil confirmed the presence of 12 metals, such as Fe, Al, Si, Na, K, B, Mo, Ni, Ca, Mg, Zn, and P (Table 1). The presence of Cu, Cr, Pb, Ag, Ti, V, Mn, Cd, and Ba was not found in any of the four tested lubricating oil samples.

The largest share in the lubricating oil samples was found to be Ca (1782 ppm), and Zn (441 ppm) and P (356 ppm) in the first tested sample. The fourth metal in terms of concentration was Boron (101 ppm). As the time of oil operation in engine increased, the concentration of these elements in oil decreased. At the same time, the presence of Ca, Mg, P, and Zn was found in deposit samples.

Elements such as Ca, P, Zn, Mg, and B are additives of lubricating oils and they improve the oils’ functional properties [17,25]. Ca is characterized by anti-wear and antioxidant properties and acts as a corrosion inhibitor and dispersant, similar to magnesium. In addition to the dispersion properties, phosphorous has analogous attributes as an addition to oils operating at extreme pressures. Zn also has anti-wear properties; it is used as an antioxidant and corrosion inhibitor [25].

An increase in the concentration of Na (from 9 to 32 ppm) and Mg was found in the oil samples over time. The presence of sodium in the lubricating oil may indicate coolant contamination; sodium salts are a component of cooling agents. After 16 weeks of engine oil operation, an increase in the concentration of Fe and Ni was observed. These elements are components of alloys used in the manufacture of engine parts. No elements such as Na, B, Mo, and Ni were found in the deposits.

On the basis of chemical analyses of sediments taken from a synthetic filter in the CMM mine, the presence of elements such as Fe, Mn, P, Zn, Cu, Cr, and As was found. The compounds that were collected in the filter included Hydrogen sulphide, Formaldehyde, Phosphine, Nitrous oxide, Acridine-9-thione, Thiophene, and Phenanthrene. The deposits were formed on the filter in the amount of about 1 kg after passing about 1 million m^3^ of CMM gas. This filter was installed directly in front of the engine to protect it.

Deposits formed on engine valves (Caterpillar Inc., Irving, TX, USA) powered by natural gas also contained elements such as Ca, S, P, and Zn, which was confirmed by the XRD technique [3]. Khan et al. [3] also analysed failed valves (polished) using SEM-EDS. Microanalysis confirmed such elements as Ni, Cr, Fe, Ti, and Al. The composition was similar with Nickel Alloy Inconel-751, which is a precipitation hardened Ni-Cr-Fe alloy, having traces of Al and Ti. This alloy is widely used in diesel engine exhaust valves. The XRD technique enabled the identification of anhydrite (CaSO_4_) as the largest share (82% by weight) of the deposits formed on the exhaust valve of a natural gas fuelled engine. The other identified compounds were hydroxyapatite (Ca_5_(PO_4_)_3_OH), zincate (ZnO), and calcite (CaCO_3_) in shares 6%, 7%, and 5%, respectively [3].

The largest differences in the composition of deposits could be observed in the case of engines fuelled by CMM and landfill gas. Deposits found in an engine powered by landfill gas contained 60–81% of Si, depending on where they were collected [29]. This element was not present in deposits collected from engine fuelled by CMM. The deposits taken from the landfill gas engine were characterized also by a lower Ca content (2–30%) compared to the deposits of CMM fuelled engines (31–51%). The average S content was also lower for landfill engine deposits. Sb (27%) was found in the deposits taken from the landfill gas combustion chamber; As also appeared—4.5% at most—as well as tin. The authors concluded that the deposits in the gas engines powered by landfill biogas were characterized by high contents of Si, Ca, P, S, and Zn [32]. These elements (Sb and As) were not found in CMM deposits (see Figure 3a, Figure 4a and Figure 5a). Östürk et al. [28] also proved that the main elements of landfill engine deposits were Si, Ca, and S, while Fe, Al, Sr, and Sn were minor elements.

Metals such as Se, Te, Hg, Pb, As, Sn, Sb, Bi, Cl, S, Mg, Cu, Si, Ca, P, and Zn have been found in analysed deposits from different parts powered by landfill gas [25,28,29,30,31]. Toxic elements such Pd, Cd, Cr, and Sb were also found in deposits on engines utilizing landfill gas; they made the deposit into potentially hazardous material [31]. In deposits of CMM fuelled engines, there was much more Zn (10–21%) when compared to landfill engine deposits (maximum of 5.8%). In CMM engine deposits, there was also about 16% of P, while it was found only in one analysed deposit in landfill engine deposits [29]. 

Anhydrite was found by Östürk et al. [28] in deposits formed on landfill-gas-fuelled engine parts such as exhaust valves, the cylinder head, and the piston head. Akaganeite (Fe^3+^O(OH)), gypsum (CaSO_4_·2H_2_O), and iron sulfate (FeSO_4_) were also found on the same parts.

For comparison, in the engine head and piston deposits from the engine fuelled with biogas produced from sewage sludge, Bi had the largest share (32–62%). Si also made up a significant share in the formation of deposits (7–24%). XRD analysis of deposits collected from various parts of the engine powered by biogas from a waste-water treatment plant confirmed oxide forms such as SiO_2_, Bi_2_O_3_, CaO, P_2_O_5_, ZnO [32].

## 4. Conclusions

The chemical composition and mass fraction determined by SEM-EDS showed that the dominant components in mineral deposits formed in our CMM gas engine were Ca, Zn, P, and S, which belonged to engine oil additives. It was noted that Al, Cr, Cu, Ti, and Fe were also present in the tested samples, which was related to the wear of the engine at normal operation. The remaining trace elements could have originated as impurities from the air or special additives derived from the oil. It is worth noting that the main source of deposit formation was engine oil in the case of the CMM engine.

The composition of the deposits formed in the combustion chambers (piston, cylinder head) was comparable due to the similar pressure and temperature conditions inside the combustion chamber. It could be assumed that the balance between the formation and removal of deposits from this part of the engine was then established.

The analysis of deposits formed in the exhaust manifold showed variability in the qualitative and quantitative composition, thus it was possible to determine which of the components of the combustion chambers were the most worn. Moreover, it was noted that the closer to the flue outlet the more diverse the deposit’s composition was. It is possible that the deposit elements’ enrichment and their composition changes occurred by exhaust gas cooling at the end of the exhaust system.

Analysis of the structure of deposits could be helpful in determining the location of excessive leakage of lubricating oil into the combustion chamber. Moreover, analysis of flue deposits could indicate the general condition of the engine.

In order to reduce the formation of deposits, care should be taken to prevent dusty particles (e.g., by using appropriate filters) and organic compounds from entering the engine. It is also important to take care of the quality of the engine’s lubricating oil, so that it does not form deposits when burned.

Further research on determining the phenomenon of deposit formation should be extended to monitoring the temperature in individual parts of the engine. Parallel to the analysis of the composition of deposits, it may be helpful in accurately determining the conditions for the formation of deposits. The second aspect will be to focus on the speciation analysis of deposits that appear on engine parts as contaminations of lubricant oil. These analyses can be crucial for development of recipes for new lubricating oils that are more resistant to contaminants in CMM. It could also enable the optimization of the engine’s operating conditions.

## Figures and Tables

**Table 1:** Elemental analysis of engine lubricating oil samples taken during operation.

Wear Metal [ppm]	Fe	Al	Si	Na	K	B	Mo	Ni	Ca	Mg	Zn	P
Sample 1 (1st week)	2	0	2	9	1	101	1	0	1782	8	441	356
Sample 2 (3rd week)	2	1	0	7	2	68	2	0	1446	13	365	306
Sample 3 (7th week)	2	0	0	10	2	69	2	2	1607	13	406	336
Sample 4 (16th week)	4	1	0	32	2	59	3	1	1621	9	375	301

## Data Availability

The data presented in this study are available on request from the corresponding author.
